# A sensitive luminescence syncytium induction assay (LuSIA) based on a reporter plasmid containing a mutation in the glucocorticoid response element in the long terminal repeat U3 region of bovine leukemia virus

**DOI:** 10.1186/s12985-019-1172-2

**Published:** 2019-05-20

**Authors:** Hirotaka Sato, Sonoko Watanuki, Lanlan Bai, Liushiqi Borjigin, Hiroshi Ishizaki, Yasunobu Matsumoto, Yuma Hachiya, Hiroshi Sentsui, Yoko Aida

**Affiliations:** 1 Nano Medical Engineering Laboratory, RIKEN Cluster for Pioneering Research, 2-1 Hirosawa, Wako, Saitama, 3510198 Japan; 2 Virus Infectious Diseases Unit, RIKEN, 2-1 Hirosawa, Wako, Saitama, 3510198 Japan; 3 Laboratory of Global Animal Resource Science, Graduate School of Agricultural and Life Sciences, The University of Tokyo, 1-1-1 Yayoi, Bunkyo-ku, Tokyo, 113-8657 Japan; 4 Grazing Animal Unit, Division of Grassland Farming, Institute of Livestock and Grassland Science, NARO, 768 Senbonmatsu, Nasushiobara, Tochigi 329-2793 Japan; 5 Department of Veterinary Medicine, Nihon University, Kameino 1866, Fujisawa, Kanagawa 252-0880 Japan

**Keywords:** Bovine leukemia virus, Infectivity, Visualization, LuSIA (luminescence syncytium induction assay), Long terminal repeat U3 region, Tax-dependent reporter, Glucocorticoid response element

## Abstract

**Background:**

Bovine leukemia virus (BLV) causes enzootic bovine leukosis, the most common neoplastic disease of cattle. Previously, we reported the luminescence syncytium induction assay (LuSIA), an assay for BLV infectivity based on CC81-BLU3G cells, which form syncytia expressing enhanced green fluorescent protein (EGFP) when co-cultured with BLV-infected cells. To develop a more sensitive LuSIA, we here focused on the glucocorticoid response element (GRE) within the U3 region of the BLV long terminal repeat (LTR).

**Methods:**

We changed five nucleotide sites of the GRE in a pBLU3-EGFP reporter plasmid containing the BLV-LTR U3 region promoter by site-directed mutagenesis and we then constructed a new reporter plasmid (pBLU3_GREM_-EGFP) in which the EGFP reporter gene was expressed under control of the GRE-mutated LTR-U3 promoter. We also established a new CC81-derived reporter cell line harboring the GRE-mutated LTR-U3 promoter (CC81-GREMG). To evaluate the sensibility, the utility and the specificity of the LuSIA using CC81-GREMG, we co-cultured CC81-GREMG cells with BLV-persistently infected cells, free-viruses, white blood cells (WBCs) from BLV-infected cows, and bovine immunodeficiency-like virus (BIV)- and bovine foamy virus (BFV)-infected cells.

**Results:**

We successfully constructed a new reporter plasmid harboring a mutation in the GRE and established a new reporter cell line, CC81-GREMG; this line was stably transfected with pBLU3_GREM_-EGFP in which the EGFP gene is expressed under control of the GRE-mutated LTR-U3 promoter and enabled direct visualization of BLV infectivity. The new LuSIA protocol using CC81-GREMG cells measures cell-to-cell infectivity and cell-free infectivity of BLV more sensitively than previous protocol using CC81-BLU3G. Furthermore, it did not respond to BIV and BFV infections, indicating that the LuSIA based on CC81-GREMG is specific for BLV infectivity. Moreover, we confirmed the utility of a new LuSIA based on CC81-GREMG cells using white blood cells (WBCs) from BLV-infected cows. Finally, the assay was useful for assessing the activity of neutralizing antibodies in plasma collected from BLV-infected cows.

**Conclusion:**

The new LuSIA protocol is quantitative and more sensitive than the previous assay based on CC81-BLU3G cells and should facilitate development of several new BLV assays.

## Background

Bovine leukemia virus (BLV), which belongs to family Retroviridae, is an oncogenic member of genus *Deltaretrovirus* that causes enzootic bovine leukosis (EBL), the most common neoplastic disease of cattle [1]. BLV infects cattle worldwide and causes serious problems for the cattle industry. For example, BLV infection decreases milk production and cow longevity without onset of leukosis [2].

BLV infectivity is typically measured using the syncytium induction assay (SIA) [3, 4]. Recently, we developed a new method for assessing BLV infectivity, the luminescence syncytium induction assay (LuSIA) [5], which uses CC81-BLU3G as the reporter cell line. CC81-BLU3G cells are stably transfected with a pBLU3-EGFP reporter plasmid harboring the BLV-LTR U3 region as the promoter and enhanced green fluorescent protein (EGFP) as the reporter gene. When these CC81-BLU3G cells are infected with BLV, they form large multinuclear syncytia that express EGFP. Thus, LuSIA facilitates detection and quantitative analysis of BLV infectivity.

The BLV long terminal repeat (LTR) consists of three regions: U3, R, and U5. The U3 region contains three Tax-responsive elements (TxREs) that are recognized by the BLV protein Tax, the main regulator of viral replication [6–8]. In particular, the binding of Tax to TxRE-2 is predominantly responsible for BLV replication [6]. Moreover, binding of Tax to BLV TxREs is mediated by the cAMP response element–binding protein (CREB) [9]. By contrast, the BLV-LTR contains multiple binding sites for several translation factors: A binding site for the interferon responding factor is present in the U5 region, and two AP-4 sites, a glucocorticoid response element (GRE), and a PU.1/Spi-B–binding site are present in the U3 region. These binding sites regulate BLV transcription, either dependent on or independently of BLV-Tax expression [7, 10–14]. However, their effects on viral replication (i.e., up- or down-regulation) differ among target cell lines [11–13]. In addition, BLV transcriptional activity is affected by acetylation and methylation of these binding sites [15, 16]. The GRE-mutated BLV-LTR promoter decreases BLV replication activity in the absence of Tax expression [11, 12] and is not affected by acetylation [14], implying that this promoter could decrease the background of BLV-LTR–derived transcription.

Here, we constructed reporter plasmids in which the EGFP reporter gene was expressed under control of the GRE-mutated LTR-U3 promoter (pBLU3_GREM_-EGFP). We also established a new CC81-derived reporter cell line harboring the GRE-mutated LTR-U3 promoter (CC81-GREMG); this line enabled direct visualization of BLV infectivity, leading to development of a more sensitive LuSIA for detection of both cell-to-cell and cell-free BLV infection. Moreover, co-culture with bovine immunodeficiency-like virus (BIV)- and bovine foamy virus (BFV)-infected cells confirmed that the LuSIA is BLV-specific. Finally, we established a new LuSIA based on CC81-GREMG cells in conjunction with white blood cells (WBCs) from BLV-infected cows. To test clinical applicability of the new assay, we examined the activity of neutralizing antibodies on plasma collected from BLV-infected cows.

## Materials and methods

### Cell cultures

FLK-BLV cells (which are persistently infected with BLV), CC81 (a feline cell line transformed by mouse sarcoma virus), and CC81-BLU3G and CC81-GREMG cells (derivatives of CC81) [5] were cultured at 37 °C in 5% CO_2_ in Dulbecco’s modified Eagle’s Medium (DMEM) (Thermo Fisher, Waltham, MA, USA) supplemented with 10% fetal bovine serum (FBS; Sigma-Aldrich, St. Louis, MO, USA). BIV-infected bovine embryonic spleen cells and BFV-infected fetal bovine muscle cells were cultured at 37 °C/5% CO_2_ in Eagle’s minimal essential medium (Nissui Pharmaceutical Co., Ltd., Tokyo, Japan) supplemented with 10% FBS, as described previously [17, 18].

### Isolation of WBCs, extraction of genomic DNA, and quantification of BLV proviral DNA

Blood samples were collected in ethylenediaminetetraacetic acid (EDTA) from breeding cows at the Institute of Livestock and Grassland Science of the National Agriculture and Food Research Organization (NARO), Tochigi Prefecture, Japan. For isolation of WBCs, red blood cells were removed from whole blood by treatment with three volumes of ACK buffer (155 mM NH_4_Cl, 10 mM KHCO_3_, 0.1 mM EDTA) for 10 min on ice. WBCs in treated blood were pelleted by centrifugation, washed once with cold phosphate-buffered saline (PBS), and then resuspended in 10% FBS/DMEM [19]. Genomic DNA was extracted from the whole blood using the Wizard Genomic DNA purification kit (Promega, Madison, WI, USA). Proviral DNA was quantified using the BLV-CoCoMo-qPCR-2 method (CoCoMo-BLV Primer/Probe; Rikengenesis, Kanagawa, Japan) as described previously [20–22].

### Construction of plasmids

pBLU3-EGFP and pME18neo/BLV-Tax-FLAG were described previously [5]. To construct pBLU3_GREM_-EGFP, mutations were inserted into pBLU3-EGFP by site-directed mutagenesis using PrimeSTAR GXL (TAKARA BIO, Shiga, Japan) and the mutagenic primer CGCATGCCAGAGATCCTATTT [11, 12].

### Establishment of reporter cells stably transfected with pBLU3_GREM_-EGFP

CC81 cells were transfected with 1.25 μg of pBLU3_GREM_-EGFP using Lipofectamine 3000. After 48 h post-transfection, the cells were cultured in 3 mL of 10% FBS/DMEM supplemented 3 μL of 50 mg/mL G418 (Roche, Basel, Switzerland), and then cultured for several weeks to amplify the antibiotic-resistant stably transfected multiple clones. Stable transfectants were cloned by limiting dilution in 96-well plates by seeding 0.8 cells/well each. The clone with the lowest background fluorescence and that responded best to transfection with 0.5 μg of pME18neo/BLV-Tax-FLAG was chosen from obtained multiple clones, and it designated CC81-GREMG.

### BLV p24 capture ELISA of FLK-BLV supernatant, including cell-free virus

FLK-BLV cells (1 × 10^6^) were seeded in 10 cm culture dishes and incubated for 3 days at 37 °C in 5% CO_2_. Culture supernatant was transferred into a 15 mL tube and centrifuged at 340×*g* for 30 min. The resultant supernatant was transferred to a 10 mL syringe (Terumo, Tokyo, Japan) and passed through a 0.3 μm pore size filter (Millipore, Darmstadt, Germany).

Cell-free virus in supernatant was quantified by BLV p24 capture ELISA [5]. Anti-BLV serum collected from a cow at the time of EBL onset and diluted 10,000-fold with carbonate/bicarbonate buffer (pH 9.6) was coated onto a 96-well ELISA plate (Beckman-Coulter, Brea, CA, USA) overnight at 4 °C. The assay plate was washed four times with PBS containing 0.05% Tween-20 (PBS-T), 200 μL of 3% skim milk in PBS was added to each well, and the plate was incubated for 1 h at room temperature. After the blocking solution was washed away, 100 μL aliquots of serially diluted FLK-BLV supernatant were applied to the wells, and the plate was incubated at 37 °C for 120 min. Anti-BLV p24 monoclonal antibody (MAb) (VMRD, Pullman, WA, USA, 1:5000), used as the detection antibody, was added and incubated for 90 min at room temperature. HRP-conjugated anti-mouse IgG (Sigma-Aldrich, 1:1000), used as the secondary antibody, was added and incubated for 60 min at room temperature. Chemi-luminescence was assessed using 1-Step Ultra TMB-ELISA (Thermo Fisher) on an EnSight plate reader (PerkinElmer, Waltham, MA, USA).

### LuSIA

CC81-BLU3G or CC81-GREMG (5 × 10^4^ cells/well) were incubated with the indicated concentration of FLK-BLV cells, serially diluted FLK-BLV supernatant, BIV-infected bovine embryonic spleen cells (5 × 10^3^ cells/well), BFV-infected fetal bovine muscle cells (5 × 10^3^ cells/well), or WBCs (5 × 10^5^, 1 × 10^5^, or 2 × 10^4^ cells/well) in LuSIA co-culture medium [DMEM supplemented with 10% FBS, 1× non-essential amino acids (Thermo Fisher), 2-mercaptoethanol (Sigma-Aldrich), 5× penicillin–streptomycin–glutamine (Thermo Fisher), and 2.5 μg/mL amphotericin B solution (Sigma-Aldrich)] in 12-well plates for 3 days. For the WBC cultures, the medium was replaced with fresh medium and the cells were cultured for an additional 2 days. The cells were washed with PBS and fixed in 3.6% formaldehyde/PBS containing 10 μg/mL Hoechst 33342 (Sigma-Aldrich). Subsequently, fluorescent syncytia in 9 or 25 fields of view in each well were automatically scanned by EVOS2 fluorescence microscopy (Thermo Fisher) with a 4× objective, and counted using the HCS Studio Cell Analysis software (Thermo Fisher) 2 days after medium change. Fluorescent syncytia were recognized by EGFP expression and gated by their area and intensity. For the BIV- and BFV-infected cell cultures, fluorescence was observed by the fluorescence microscopy (Model IX71S1F-3, Olympus corporation, Tokyo, Japan) fitted with 10× objective lens.

### BLV neutralization assay

Blood samples were collected (in tubes containing EDTA) from a BLV-uninfected cow and from a BLV-infected cow at the time of EBL onset. Samples were centrifuged at 340×*g* for 30 min, and the clear supernatant was collected as plasma. Plasma samples were serially 2-fold diluted with LuSIA co-culture medium. CC81-GREMG cells were seeded at 5 × 10^3^ cells/well in 100 μL of LuSIA co-culture medium in 96-well plates. To each well was added 50 μL of diluted serum containing 1 × 10^3^ FLK-BLV cells, and the samples were co-cultured at 37 °C for 24 h. Fluorescent syncytia were counted as described above.

## Results

### Establishment of a new reporter plasmid containing the mutant form of the GRE within the U3 region of the BLV-LTR

**Fig. 1:**
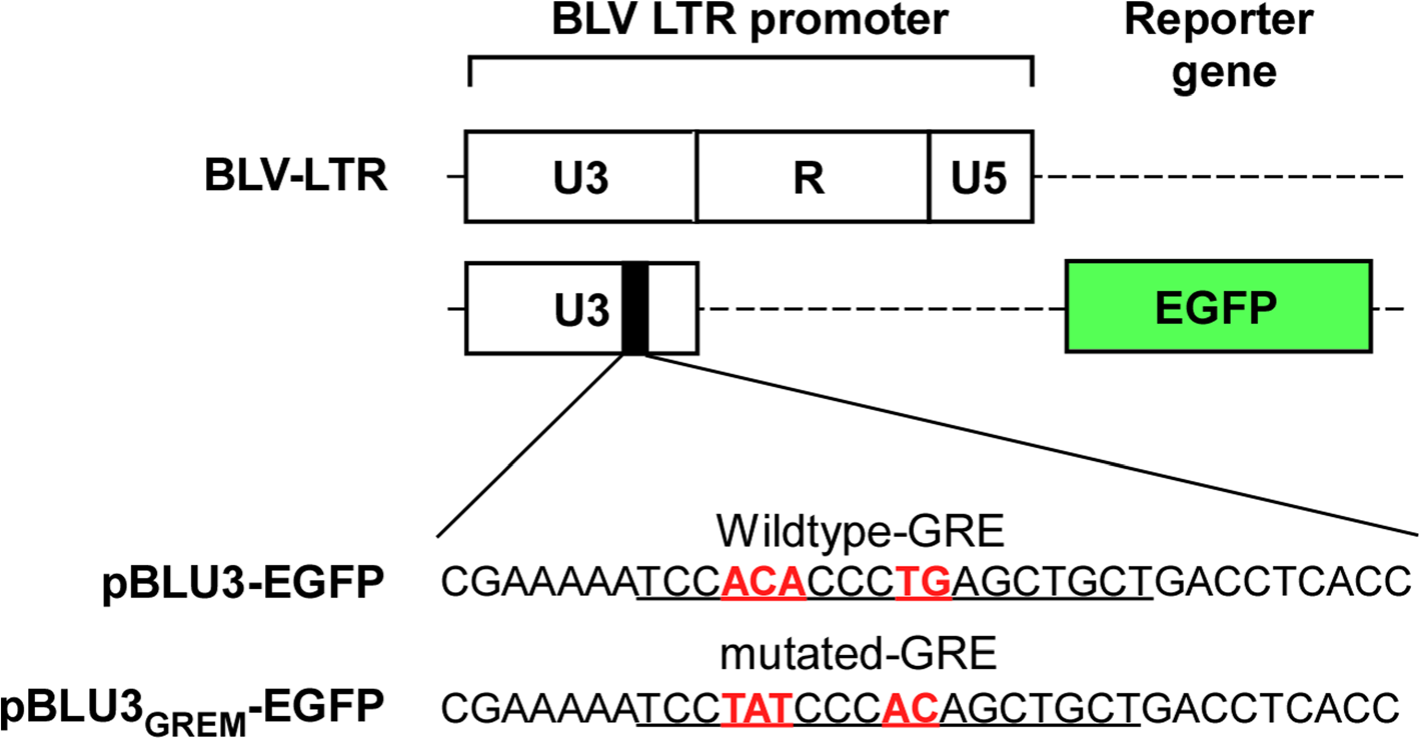
Construction of reporter plasmids. Schematic presentation of reporter plasmids bearing several BLV-LTR promoters: the full-length BLV-LTR promoter; pBLU3-EGFP with the BLV-LTR U3 region promoter; and pBLU3_GREM_-EGFP with a BLV-LTR U3 region containing a mutation in the glucocorticoid response element (GRE). The sequence of the GRE in the BLV-LTR U3 region and its inactive mutation were reported previously [11, 12]

Previously, we established a reporter cell line, CC81-BLU3G, and used it to measure the cell-to-cell infectivity of BLV-infected cells; the cell line harbors a reporter plasmid, pBLU3-EGFP, which contains the BLV-LTR U3 region linked to the EGFP gene [5]. To develop more sensitive assays for BLV infectivity, we tried to establish more sensitive fluorescence-based reporter cell lines. A previous report shows that the GRE in the LTR U3 region is involved in translational activity independent of *tax* activation [14]. We considered that reporter cells containing a reporter plasmid harboring a GRE-mutated LTR-U3 promoter will be more sensitive due to reduced background fluorescence. Hence, we constructed a new reporter plasmid (pBLU3_GREM_-EGFP) harboring a mutant form of GRE in the U3 region (Fig. 1).

### Establishment of a new reporter cell line and evaluation of its utility as a reporter for cell-to-cell infection by LuSIA

**Fig. 2:**
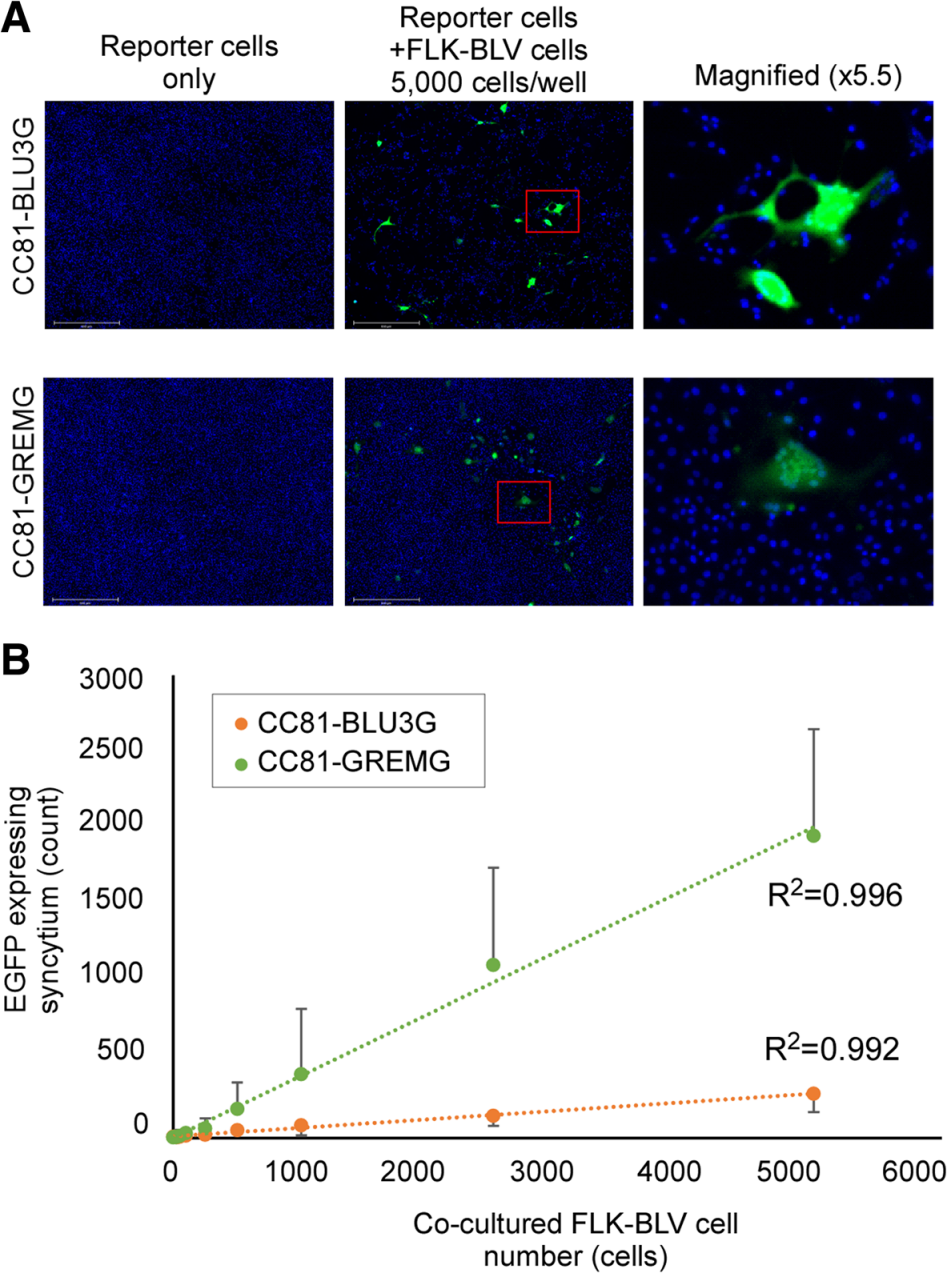
Comparison of quantitative analysis of cell-to-cell infection by LuSIAs using CC81-GREMG and CC81-BLU3G reporter cells. To compare the sensitivity of the newly developed reporter cell line CC81-GREMG, which is stably transfected with pBLU3_GREM_-EGFP, and the previously developed reporter cell line, CC81-BLU3G, the cells were cultured with FLK-BLV cells, which are productively infected by BLV. **a** CC81-GREMG or CC81-BLU3G was cultured with or without FLK-BLV cells. **b** Correlation of the number of EGFP-expressing syncytia and FLK-BLV cell number, when CC81-BLU3G or CC81-GREMG cells were co-cultured with serially diluted FLK-BLV cells. Results indicate the mean and standard deviation of three independent experiments

To generate a more sensitive reporter cell line, pBLU3_GREM_-EGFP was transfected into CC81 cells and stable transfectants were obtained; the resultant cells were named CC81-GREMG. CC81-GREMG formed EGFP-expressing syncytia when co-cultured with FLK-BLV cells, which constitutively express BLV (Fig. 2a).

To evaluate the sensitivity of CC81-GREMG, we performed LuSIA by co-culturing CC81-GREMG or CC81-BLU3G cells with 0, 156, 312, 625, 1250, 2500, or 5000 FLK-BLV cells/well. In LuSIAs using both reporter cell lines, the number of fluorescence syncytia was strongly correlated with the number of FLK-BLV cells (R^2^ = 0.996 for CC81-GREMG; R^2^ = 0.992 for CC81-BLU3G) (Fig. 2b). Fortunately, the fluorescent syncytia numbers obtained by LuSIA were higher when using CC81-GREMG (5 ± 4 to 1961 ± 632 counts/well) than CC81-BLU3G (9 ± 8 to 288 ± 66 counts/well), suggesting that CC81-GREMG is more suitable for analyzing cell-to-cell infectivity of BLV by LuSIA.

**Fig. 3:**
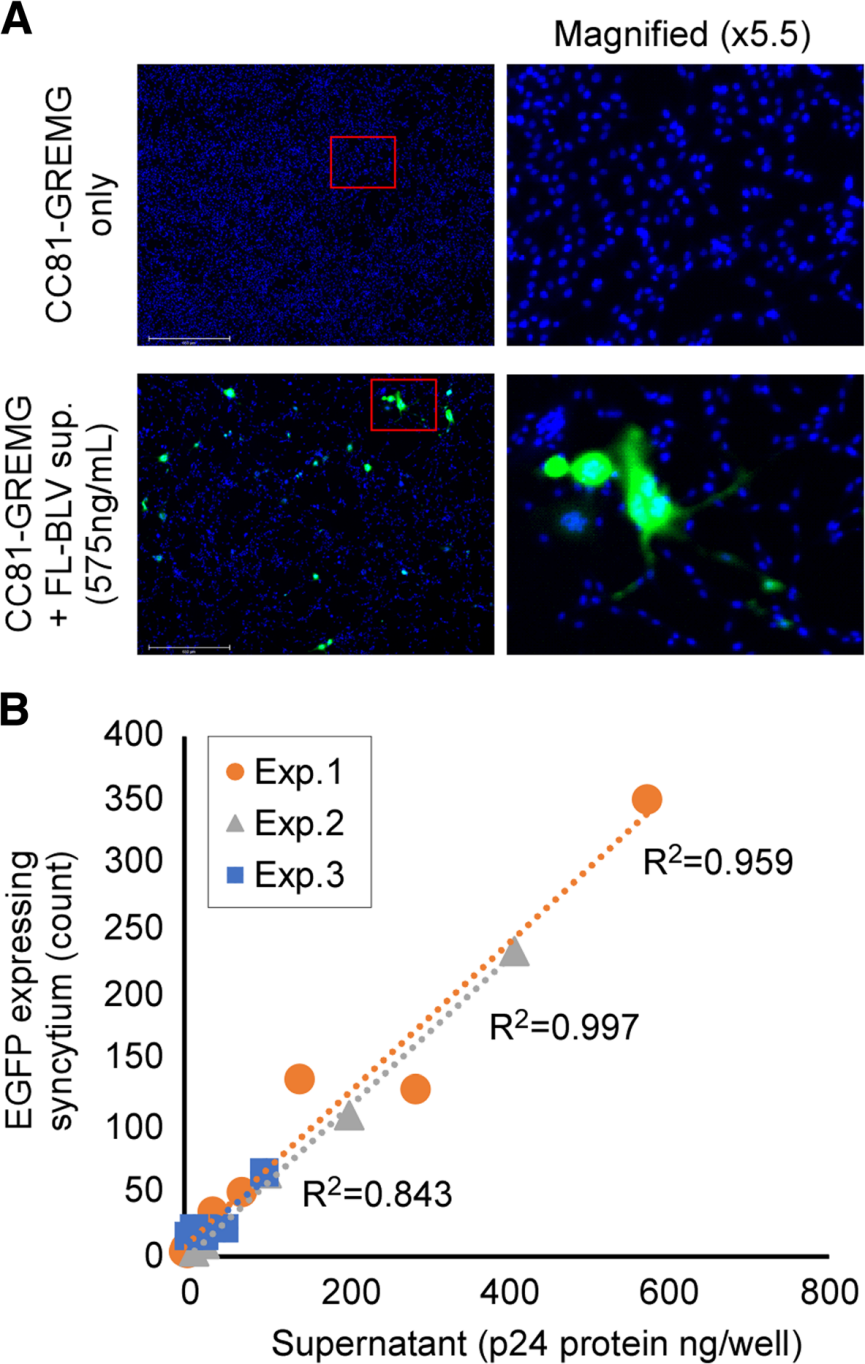
Quantitative analysis of cell-free infection by LuSIA using CC81-GREMG. **a** CC81-GREMG cells were cultured with or without supernatant collected from FLK-BLV cells, which are productively infected with BLV. **b** Correlation of the amount of BLV p24 protein ELISA in the culture supernatant, as determined by BLV p24 capture, and the number of syncytia expressing EGFP when CC81-GREMG cells were cultured with serially diluted FLK-BLV supernatant. Results of three independent experiments are shown individually

Next, we attempted to use CC81-GREMG cells to detect cell-free infection of BLV. To this end, CC81-GREMG was infected with culture supernatant collected from FLK-BLV cells. The FLK-BLV supernatant induced fluorescent syncytia on the CC81-GREMG cells (Fig. 3a) to an extent that was highly dependent on the level of BLV p24 protein, as determined by BLV p24 capture ELISA (Fig. 3b).

### The LuSIA based on CC81-GREMG does not respond to other syncytium-inducible viruses

**Fig. 4:**
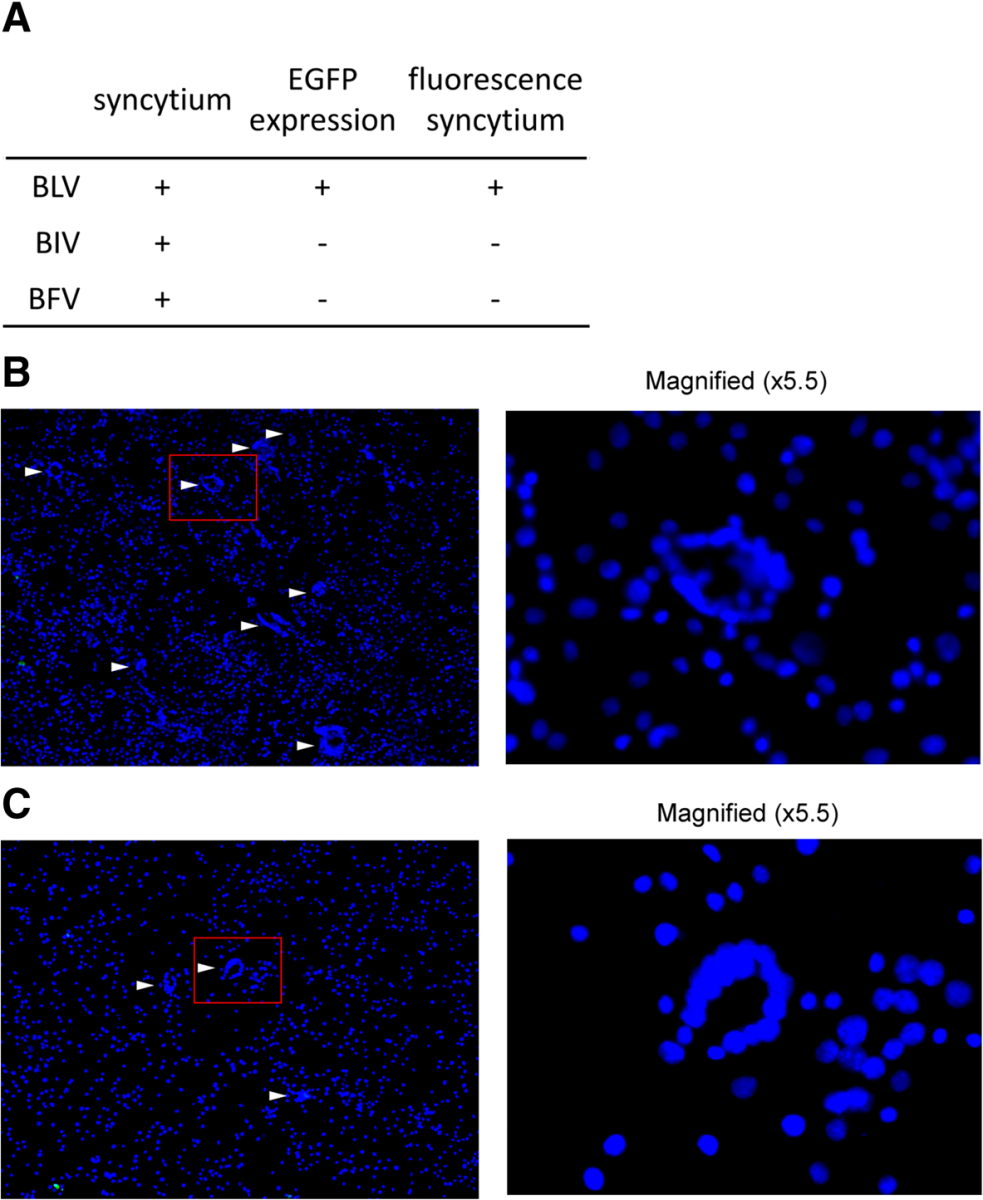
Use of LuSIA based on CC81-GREMG cells to detect other syncytium-inducible bovine viruses. **a** Specificity of the LuSIA. Data are representative of three independent experiments. CC81-GREMG cells were co-cultured with bovine immunodeficiency-like virus (BIV)-infected bovine embryonic spleen cells (**b**), and with bovine foamy virus (BFV)-infected fetal bovine muscle cells **c**

To confirm whether the LuSIA detects BLV-specific infections, we co-cultured CC81-GREMG cells with BIV- and BFV-infected cells. BIV- and BFV-infected cells, which were established as reported previously [17, 18], induced syncytium formation when co-cultured with CC81 cells. When CC81-GREMG cells were co-cultured with cells infected with BIV (Fig. 4b) or BFV (Fig. 4c), they showed syncytium-like formation; however, they did not emit green fluorescence derived from EGFP expression or show fluorescent syncytia when co-cultured with BLV-infected cells. These results indicate that the LuSIA based on CC81-GREMG is specific for BLV infectivity (Fig. 4a).

### Establishment of a CC81-GREMG–based LuSIA using WBCs from BLV-infected cows

**Fig. 5:**
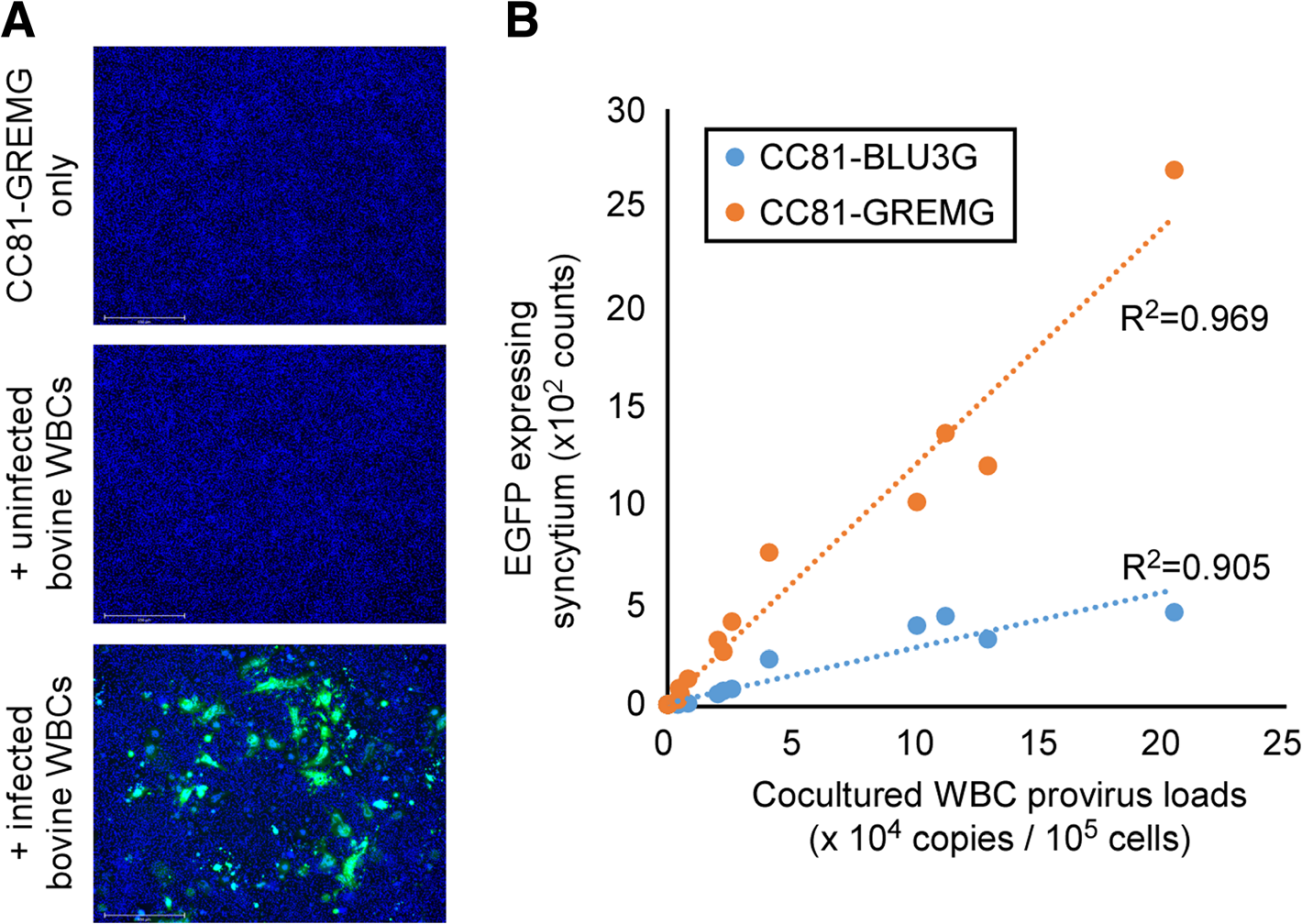
Comparison of LuSIAs using CC81-GREMG and CC81-BLU3G with white blood cells (WBCs) from BLV-infected cows. Infectivity of BLV was assessed by LuSIA using CC81-GREMG with 5 × 10^5^, 1 × 10^5^, or 2 × 10^4^ WBCs collected from seven BLV-infected and two uninfected cows. **a** CC81-GREMG cells were cultured with or without 1 × 10^5^ WBCs collected from BLV-infected or -uninfected cows. **b** Correlations between BLV provirus load and the number of syncytia expressing EGFP, when CC81-BLU3G and CC81-GREMG cells were co-cultured with WBCs. Provirus loads were assessed by the CoCoMo-qPCR-2 method

Previously, we reported that the BLV proviral load correlates strongly with the number of syncytia and the number of syncytia expressing EGFP, as measured by SIA and LuSIA based on CC81-BLU3G cells [5, 20]. To develop a new LuSIA using CC81-GREMG that could be adapted to samples collected from BLV-infected cows, we co-cultured CC81-BLU3G or CC81-GREMG cells along with 5 × 10^5^, 1 × 10^5^, or 2 × 10^4^ WBCs collected from nine cows. CC81-GREMG cells responded to WBCs collected from seven BLV-infected cows, but not those from two uninfected cows (Fig. 5a). LuSIA using CC81-GREMG yielded a higher correlation between BLV provirus load and the number of syncytia expressing EGFP (R^2^ = 0.969) than LuSIA using CC81-BLU3G (R^2^ = 0.905), and fluorescent syncytia numbers were more abundant in the former (9–2695 counts/well) than in the latter (9–472 counts/well), as in co-culture with FLK-BLV cells (Fig. 5b).

**Fig. 6:**
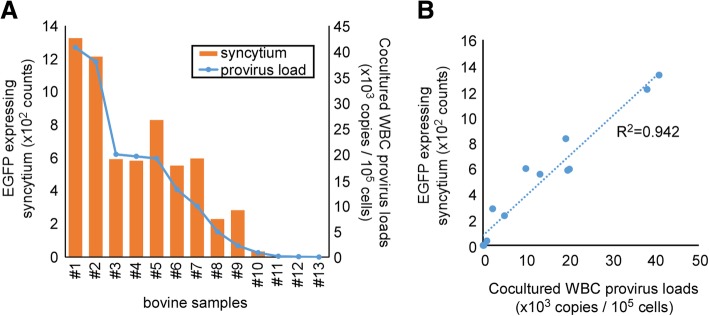
LuSIA using CC81-GREMG with white blood cells (WBCs) from BLV-infected cows. **a** WBCs (1 × 10^5^ cells) collected from 12 BLV-infected cows and one uninfected cow were cultured with CC81-GREMG for 3 days. **b** The correlation between provirus copy number and the number of fluorescent syncytia is shown. Provirus loads were assessed by the CoCoMo-qPCR-2 method

To confirm the utility of LuSIA using CC81-GREMG, we collected and analyzed 13 more blood samples from the same farm (Fig. 6a). As shown in Fig. 6b, the number of syncytia expressing EGFP was highly correlated to the BLV provirus loads (R^2^ = 0.942).

### Application of LuSIA with CC81-GREMG to BLV antibody neutralization assay

**Fig. 7:**
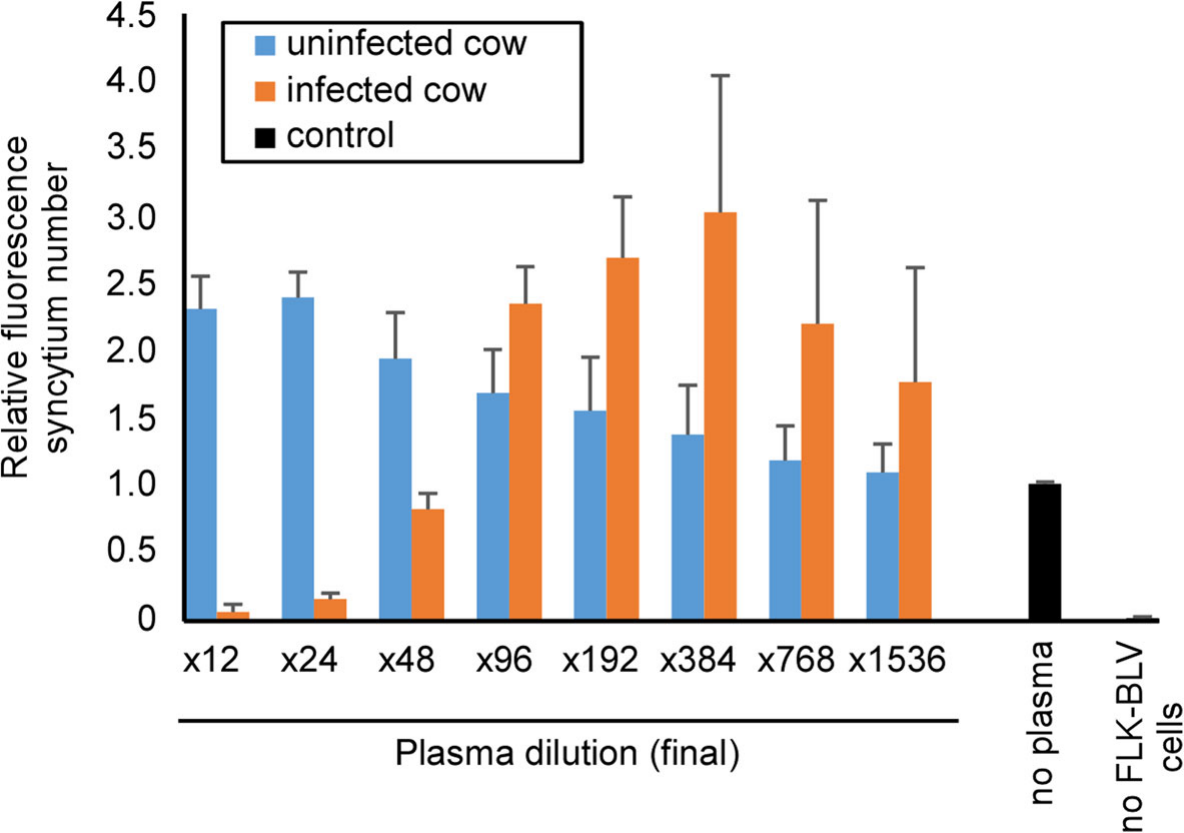
Neutralization assay using plasma taken from BLV-infected cows by LuSIA using CC81-GREMG. CC81-GREMG cells were co-cultured with FLK-BLV cells in the presence of diluted plasma collected from BLV-infected cows with lymphoma (red) or uninfected cows (blue). CC81-GREMG cells co-cultured with FLK-BLV cells in the absence of plasma were used as negative controls, and cultures of CC81-GREMG cells alone were used as positive controls. Results show the mean and standard deviation of two independent experiments

As a clinical application of LuSIA with CC81-GREMG, we developed an antibody neutralization assay using this method. In these experiments, CC81-GREMG cells were co-cultured with FLK-BLV cells in the presence (dilutions: 12-, 24-, 48-, 96-, 192-, 384-, 768-, or 1536-fold) of plasma collected from BLV-infected cattle (Fig. 7). The number of fluorescent syncytia was quite low at high concentrations of serum from BLV-infected cattle (12-, 24-, or 48-fold dilutions), but was higher in the presence of the same concentrations of serum collected from BLV-uninfected cattle. This result shows that LuSIA using CC81-GREMG is useful for assessing the neutralization activity of antibodies in serum.

## Discussion

The BLV-LTR U3 region plays a critical role in regulating BLV-Tax–dependent viral replication. Previously, we reported a method for BLV infectivity analysis, which we named the LuSIA [5]. This assay uses the established reporter cell line CC81-BLU3G, stably transfected with a reporter plasmid in which the BLV-LTR U3 region serves as the promoter. LuSIA with CC81-BLU3G cells could measure the cell-to-cell BLV infectivity clearly and quantitatively. However, the BLV-LTR U3 region also contains other transcription factor response elements that regulate viral replication [7, 10–16]. Therefore, in this study, we constructed a new plasmid, which is a variant of the BLV-LTR U3 promoter harboring a mutation in the GRE sequence (Fig. 1). The mutated GRE promoter generated a significantly weaker background signal than promoters harboring the wild-type GRE sequence, resulting in a higher signal-to-background ratio (data not shown). This result suggested that reporter cells bearing a GRE-mutated U3 promoter would be more suitable for our BLV infectivity assay than previously established cell lines. Accordingly, we established a stably transfected cell line called CC81-GREMG. CC81-GREMG cells expressed EGFP efficiently in response to co-culture with FLK-BLV cells, which constitutively express BLV (Fig. 2a), and as expected, they also exhibited lower background fluorescence. Additionally, significantly more syncytia expressing EGFP were detected in assays using CC81-GREMG than in those using our previously established cell line, CC81-BLU3G (Fig. 2b). The reduction in background fluorescence made a major contribution to identification of samples with lower infectivity, likely because the decrease in background fluorescence made it easier to detect fluorescent syncytia with lower intensities.

Because LuSIA using CC81-GREMG was more sensitive, we attempted to use it to measure cell-free BLV infectivity. BLV was naturally and mainly transmitted via cell-to-cell but not cell-free, because it is extremely lower extent and less their infectivity of virions in the blood of BLV-infected cattle [23]. In addition, it is considered that cell-free infection occurs only limited artificial condition like a vaccination using culture supernatant of infected cell lines. Therefore, we used culture supernatant from FLK-BLV cells bearing infectious BLV virions, which is sufficient to infect the reporter cell line [24]. When CC81-GREMG cells were cultured with FLK-BLV culture supernatant containing virus particles, we successfully observed EGFP-expressing syncytia (Fig. 3a). Thus, we could quantitatively assess BLV cell-free infectivity, which was clearly correlated with the amount of BLV p24 protein in the supernatant (Fig. 3b).

Co-culture of BIV and BFV with CC81 cells results in syncytium formation (as observed for BLV) [17, 18]. Therefore, we confirmed that the LuSIA based on CC81-GREMG cells is specific for BLV (Fig. 4). CC81-GREMG expressed EGFP in the presence of the BLV viral transcriptional protein Tax, but not in the presence of BIV Tat and BFV Tas proteins, suggesting that LuSIA based on CC81-GREMG cells is highly specific for BLV. This result is very important when assays of BLV infectivity are carried out on farms with multiple/mixed infections. Indeed, mixed infection of a single cow with BLV and other viruses has been reported [25]; Recently, we constructed luciferase-based reporter plasmids pBLU3-Luc and pBLU3_GREM_-Luc, which have no requirement for syncytium formation to assess the BLV infectivity, and established stably transfectant cell lines CC81-BLU3L and CC81-GREML, respectively (unpublished data). This appears to be one way of solving the problem of BLV infectivity assay on the mix-infected cow and will be a useful tool experimentally; however, it is expensive and more complicated than the system based on EGFP visualization. Taken together, these results suggest that LuSIA based on CC81-GREMG is highly sensitive and specific, and as such will be useful for developing analytical methods such as titration of anti–BLV-neutralizing antibodies.

In this study, we clearly demonstrated that our new LuSIA with CC81-GREMG is more useful than LuSIA with CC81-BLU3G for measurement of BLV infectivity. In our previous report, we used peripheral blood mononuclear cells as the infected bovine samples for co-cultivation; however, WBCs are easier to isolate and can also be used for co-cultivation (data not shown). Therefore, to confirm that our new protocol for LuSIA using CC81-GREMG could be used to assess the BLV infectivity of infected cows, we co-cultured CC81-GREMG with WBCs collected from BLV-infected cows. LuSIA using the CC81-GREMG protocol could also detect BLV infectivity (Fig. 5a), and as expected had higher resolution than our previous protocol using CC81-BLU3G (Fig. 5b). Furthermore, the number of syncytia expressing EGFP was strongly correlated with BLV provirus loads (Fig. 6b).

We also successfully assessed the titer of neutralizing antibodies in plasma collected from BLV-infected cows (Fig. 7). The plasma which collected from BLV-infected cattle includes the specific antibodies toward against BLV viral proteins, but not virions itself [23]. We could clearly discriminate the plasma of BLV-infected cows, which has BLV-neutralizing activity, from the plasma of uninfected cows. The number of fluorescence syncytia caused by co-culture with FLK-BLV was clearly decreased by plasma collected from BLV-infected cows (at 12–48-fold dilutions). By the way, it increased by presence of 12- to 768-fold diluted plasma collected from BLV-uninfected cow and over 96-fold diluted plasma collected from BLV-infected cow compared with control wells without plasma. This phenomenon suggests that plasma act as a nutrient, analogous to FBS, for CC81-GREMG and/or FLK-BLV cells. Accordingly, this result suggests that LuSIA using the CC81-GREMG protocol can not only be used to assess infectivity, but could also be adapted to other assays.

## Conclusion

We developed a new protocol for LuSIA using CC81-GREMG cells to measure BLV infectivity. The new method is quantitative and more sensitive than the previous protocol using CC81-BLU3G cells. The new LuSIA is adaptable to several types of assay, such as BLV neutralization by plasma or serum, BLV contamination contradiction assay of bovine vaccines, and screening of anti-viral drugs.

## Abbreviations

BFV: Bovine foamy virus
BIV: Bovine immunodeficiency-like virus
BLV: Bovine leukemia virus
CREB: CAMP response element–binding protein
DMEM: Dulbecco’s modified Eagle’s Medium
EBL: Enzootic bovine leukosis
EDTA: Ethylenediaminetetraacetic acid
EGFP: Enhanced green fluorescent protein
FBS: Fetal bovine serum
GRE: Glucocorticoid response element
LTR: Long terminal repeat
LuSIA: Luminescence syncytium induction assay
MAb: Monoclonal antibody
PBS: Phosphate-buffered saline
PBS-T: PBS containing 0.05% Tween-20
SIA: Syncytium induction assay
TxRE: Tax-responsive elements
WBCs: White blood cells
